# Maternal Factors Affecting the Macronutrient Composition of Transitional Human Milk

**DOI:** 10.3390/ijerph19063308

**Published:** 2022-03-11

**Authors:** Chung Ja Ryoo, Nam Mi Kang

**Affiliations:** Department of Nursing, Research Institute for Biomedical & Health Science, Konkuk University, Chungju 27478, Chungcheongbuk-do, Korea; rcj1109@hanmail.net

**Keywords:** breastfeeding assessment, dietary intake of macronutrients, human milk macronutrients, postpartum stress, sleep quality, spousal support

## Abstract

This study investigated the influence of selected maternal factors on the macronutrient composition and energy of human milk (HM). The study enrolled 159 breastfeeding mothers from five postpartum care centers in Seoul, Korea. Their gestational weeks were 37–42 weeks, they had no complications before and after childbirth, and were at 7–14 days postpartum. They provided data using structured questionnaires on general characteristics, stress, sleep quality, spousal support, and dietary intake. Breastfeeding assessment (LATCH) was investigated by qualified nurses, and each mother provided one sample of HM. The HM composition was analyzed using the Miris^®^ HM analyzer. The relationships between variables were analyzed using Pearson’s correlation analysis, and a linear regression analysis was performed to verify the main variables. It was found that maternal dietary intake was related to HM composition as the %energy from carbohydrates (β = 0.86, *p* < 0.01) and %energy from fat (β = 0.77, *p* < 0.05) showed positive relationships with HM energy. The LATCH score was positively related to HM energy (β = 0.17, *p* < 0.05). In contrast, postpartum stress, sleep quality, and spousal support were not associated with HM macronutrient composition. In conclusion, HM macronutrients and energy content were associated with maternal dietary intake and LATCH scores, but not with postpartum stress, sleep quality, and spousal support.

## 1. Introduction

Human milk (HM) is an optimal source of nutrition for the growth and development of babies. It is made up of very diverse and complex ingredients. That is, macronutrients such as carbohydrates (lactose), proteins, fats, and various vitamins and minerals, as well as various bioactive factors, immunoglobulins, metabolites, and microorganisms that play specific physiological roles are included in the HM. Hence, breastfeeding may provide the best start to life, offering long-term and short-term health, nutritional and emotional benefits to mothers and babies [1]. Furthermore, breastfeeding is positively associated with the enhancement of human capital, including improved performance in intelligence tests, greater academic achievement, and higher income compared to formula-fed infants [2].

The composition of HM is dynamic and varies according to the factors affecting it. Characterizing HM with precision requires an understanding of the factors that modulate its composition. It has been reported that HM composition is affected by lactation periods, maternal body mass index (BMI), ethnicity, environment, lifestyle, etc. For example, He et al. reported that as lactation progressed from 0–5 to 15–30 days, the lipid content increased significantly, whereas the protein concentration and the proportions of phospholipids and cholesterols decreased notably [3]. Another study showed that maternal BMI and age affected fatty-acid levels in HM [4,5], and maternal obesity was associated with changes in the HM metabolome [6]. In the case of ethnicity, Bruun et al. reported that the median osteopontin concentration in HM varied across sites; from 99.7 mg/L in Danish to 266.2 mg/L in Chinese mothers (*p* < 0.001), corresponding to 1.3%, and 2.7% of the total protein content (OPN/protein%) (*p* < 0.05), respectively [7]. Nevertheless, the role of each of these influences has not been well defined for the many HM components, and it is not clear how variable or consistent the HM composition is across populations [8].

Maternal diet and nutritional status have also been studied in terms of their effect on the HM composition. Some studies reported significant effects of fat concentration on the HM composition [9,10]. However, various studies reported no significant association between diet and HM composition. For instance, a study showed that breastfeeding mothers’ diet generally did not affect HM macronutrient components [11], and even if a breastfeeding mother did not consume the recommended daily calories or nutrients, the quantity of HM or macronutrients such as carbohydrates, proteins, fats, and calories and the content of major minerals such as calcium were not affected [12,13] As such, the results of studies on dietary factors for HM components are mixed.

Mothers may go through various psychosocial changes after giving birth. They may experience negative emotional changes such as stress in the process of adjusting to the new role as a mother [14]. Immediately after childbirth, changes in appearance and body shape, physical discomfort such as breast engorgement, lack of sleep, eating disorders, difficulties in breastfeeding, insufficient milk supply, and lack of support from spouses were considered high stress factors during the postpartum period [15,16,17]. In addition, stress or poor quality of sleep in breastfeeding mothers can lead to abnormal nutritional metabolism due to changes in dietary intake or energy consumption [18]. Depressive symptoms, stress, and sleep quality were associated with reward-related eating during postpartum [19], and postpartum health-provider support was associated with diet quality [20].

In responding to the various difficulties of the postpartum period, Tornese et al. suggested that breastfeeding assessment could help ameliorate difficulties caused by breastfeeding [21], and Kivijarvi et al. reported that spousal support was effective for psychological stability and postpartum adjustment in breastfeeding mothers [22]. Contrarily, insufficient spousal support led to high levels of stress in breastfeeding mothers [23].

These studies show that both maternal psychosocial factors and maternal lifestyle in postpartum may directly or indirectly affect HM components. Hence, this study pays attention to these factors in identifying the variables associated with components of transitional HM.

In terms of the relationship of psychosocial factors with HM, some studies report effects of stress and anxiety on HM such as stress leading to inadequate milk volume [24]. However, other studies show that they do not meaningfully affect the HM macronutrient composition [25], nor the baby’s intestinal flora [26]. In the case of Soffer et al., who looked at the effects on HM macronutrient composition, their study targeted participants from a limited regional scope and only considered potential effects of anxiety. Therefore, there is further need to investigate other psychosocial and lifestyle factors of breastfeeding mothers, as well as a need to check possible differences in results for breastfeeding mothers from distinct ethnicity and regional groups. Furthermore, the study of Soffer et al. [25] had only 21 participants, so additional research is required to expand the number of participants to confirm the reliability of results.

In this sense, the purpose of this study is to identify factors affecting the HM macronutrients and energy of breastfeeding mothers in Korea, examining postpartum stress, sleep quality, spousal support, and dietary intake of macronutrients and breastfeeding assessment (LATCH) in breastfeeding mothers who are at 2 weeks postpartum. This will provide meaningful empirical foundations for developing dietary and breastfeeding education programs to promote breastfeeding, adaptation and psychological breastfeeding interventions for breastfeeding mothers in their transition periods, as well as provide basic data for setting standards for HM components.

## 2. Materials and Methods

### 2.1. Study Design

This cross-sectional study is a descriptive observational research to identify the effects of psychosocial and lifestyle factors of breastfeeding mothers at 2 weeks postpartum on the macronutrients and energy of HM.

### 2.2. Participants

The study participants who were breastfeeding mothers, were enrolled in the study during February 2021 to April 2021 at postpartum care centers located in Seoul, Korea. Participants of the study were recruited based on the following inclusion criteria: gestational weeks between 37 to 42 weeks; 18 to 45 years old; no complications before and after childbirth; no acute and chronic health problems for both the mother and baby; and from 7 to 14 days postpartum. All were single pregnancies. Among the 200 mothers initially enrolled in the study, 159 of them became the final study participants after eliminating 30 incomplete surveys, 10 data outliers, and 1 absent HM sample.

The sample size was calculated based on the model provided in Min et al. [27] analyzing HM components, and the statistical power of multiple linear regression calculated by G*power was 0.99.

### 2.3. Data Collection

#### 2.3.1. Structured Questionnaire Survey for Health Information

The maternal information on general characteristics (age, parity, employment status, breastfeeding education, feeding type, BMI, etc.) and main variables of participants including postpartum stress, sleep quality, and spousal support were collected through structured questionnaires. The 7th Food Intake Frequency Survey [28] questionnaire was used to investigate the participants’ dietary intake of macronutrients. This questionnaire consists of responses to how often Koreans have eaten 112 favorite foods in the past year (number of intakes/month, or week, or day) and average intakes at a time with a comparable standard amount. The frequency and amount of food intake of the breastfeeding mothers were calculated as the ratio of energy intake from carbohydrates, energy ratio from protein, and energy intake from fat, which are three sub-items of macronutrient intake. Breastfeeding assessment score was obtained by Jensen’s LATCH breastfeeding assessment tool [29].

#### 2.3.2. Human Milk Sample Collection and Analyses

Transitional HM samples were collected manually. In order to maintain same conditions for collecting HM samples among lactating mothers to the extent possible, the mothers were asked to collect HM from 7 to 14 days after childbirth between 1:00 PM and 5:00 PM after breastfeeding. Principally, the mothers were requested to collect 10–30 mL of HM in a sterile plastic bag using a breast pump. Immediately after extraction, samples were put in a freezer at −20 °C or below prior to transfer via fast frozen delivery service to Konkuk University laboratory and were pre-divided into an amount required for analysis and stored at −80 °C until thawing for analysis. Each frozen sample was slowly heated near 37 °C using a constant temperature water bath (DS-DWB22, Dongseo Science, Seoul, Korea), then homogenized using a milk homogenization device (MIRIS^®^ Sonicator) before use. For homogenized HM at 37 °C, carbohydrate, protein, fat content, and energy were measured by putting 1mL of HM into the analyzer for 1 min using the Miris^®^ HM analyzer (Miris holding, Uppsala, Sweden). All samples were measured and used for analysis by the trained researchers. The donated HM that had been analyzed for this research was immediately discarded right after the research, and if the lactating mother agreed in writing to the use of her HM sample for further research, it was stored in the freezer at below −80 °C in the laboratory of Konkuk University for up to 2 years after the completion of the study.

### 2.4. Data Analysis

Data were analyzed using SPSS version 20.0 and an R Language-based JAMOVI program for graphics with statistical significance set at *p* < 0.05. For data refinement, normality was checked through skewness and kurtosis values. For variables whose normality was not secured, a box plot was derived, and samples with response values greater than 1.5 times the IQR (interquartile range) were identified as outliers and were removed. The general characteristics, postpartum stress level, sleep quality, spousal support, and dietary intake of macronutrients, and breastfeeding assessment were analyzed using descriptive statistics. The relationships between variables were analyzed using the Pearson’s correlation coefficient. A regression analysis using a backward method was performed to verify the main variables presented in this study, with HM macronutrient components as the dependent variable and postpartum stress, sleep quality, spousal support, dietary intake of macronutrients, and breastfeeding assessment as the independent variables. In examining the effects of the independent variables on the dependent variable, the general characteristics of the study participants were controlled for. In addition, the regression analysis was performed in a backward manner, meaning insignificant independent variables in terms of their association with the dependent variable were removed in the identification process of variables with significant influence. The model was continuously modified to obtain the final model.

### 2.5. Ethics

This research was approved by the Institutional Review Board of the Konkuk University (No. 7001355-202102-HR-420) and written informed consent in accordance with the Declaration of Helsinki was obtained from the respondents who agreed to participate in the study.

## 3. Results

### 3.1. General Characteristics, Postpartum Stress, Sleep Quality, Spousal Support, and Dietary Intake of Macronutrients, Breastfeeding Assessment, HM Macronutrients and Energy

The results of frequency analysis and descriptive statistical analysis conducted to examine the characteristics including personal background, childbirth background, lactation background, and BMI of the study participants with the data of 159 people are shown below.

The mean age of all 159 participants was 33.98 ± 4.35 years. Of the total participants, 66.7% had one childbirth experience, and the type of delivery was natural childbirth in 54.7%. As for the type of lactation, mixed feeding was 79.9%, and 72.3% had no experience in breastfeeding education. The current BMI was found to be 24.16 kg/m^2^ ± 2.73 on average. Further details on the general characteristics are reported below (Table 1).

Descriptive statistics of the main variables used in this study are shown in Table 2 below.

The mean scores of postpartum stress, sleep quality, and spousal support were 18.54 ± 4.21 (range: 9–27), 55.81 ± 18.37 (range: 13–97), and 105.56 ± 15.97 (range: 25–125), respectively. The average ratio of energy consumed from carbohydrates, protein, and fat of macronutrient intake was 57%E ± 0.10 (range: 35–79%E), 15.9%E ± 0.04 (range: 9–28%E) and 26%E ± 0.07 (range: 10–42%E), respectively. In addition, the mean score of the breastfeeding assessment was 7.55 ± 1.84. The mean scores of HM carbohydrates, protein, fat component, and energy were 6.64 g/dL ± 0.27 (range: 5.70–7.40 g/dL), 1.32 g/dL ± 0.25 (range: 0.50–2.40 g/dL), 3.45 g/dL ± 1.28 (range: 0.80–7.90 g/dL), and 63.18 kcal/dL ± 11.22 (range: 33.00–102.00 kcal/dL), respectively.

In examining normality, the skewness was at most −1.30 or less and the kurtosis was at most 3.31 or less, indicating that normality was secured.

### 3.2. Correlation Analysis of Postpartum Stress, Sleep Quality, Spousal Support, Dietary Intake of Macronutrients, Breastfeeding Assessment, and HM Macronutrients and Energy

In the correlation analysis, postpartum stress was found to have a significant positive relationship with sleep quality (r = 0.34, *p* < 0.01) and significant negative correlations with spousal support (r = −0.35, *p* < 0.01) and breastfeeding assessment (r = −0.21, *p* < 0.01). The sleep quality score showed a significant negative correlation with spousal support (r = −0.16, *p* < 0.05). A higher sleep quality score indicates lower sleep quality, which means the higher the stress, the lower the sleep quality, and higher spousal support and breastfeeding assessment scores indicate better sleep quality. Spousal support had a significant positive relationship with breastfeeding assessment (r = 0.26, *p* < 0.01), but it showed a significant negative correlation (r = −0.16, *p* < 0.05) with the ratio of energy intake from protein in macronutrient intake. The ratio of energy intake from protein of macronutrient intake showed a significant negative correlation with HM fat content (r = −0.17, *p* < 0.05) and HM energy (r = −0.22, *p* < 0.05). Also, breastfeeding assessment showed a significant positive correlation with HM energy (Table 3).

### 3.3. Maternal Factors Affecting Energy and Macronutrient Composition of Transitional HM

The linearity and independence of residuals were confirmed as a result of checking the Q–Q plot and residual plot of the final model of the regression model between maternal factors and HM carbohydrate, protein, fat, and energy.

As a result of examining the effects of postpartum stress, sleep quality, spousal support, dietary intake of macronutrients, and breastfeeding assessment on the energy and macronutrient composition of transitional HM, no significant associations were found between postpartum stress, sleep quality, and spousal support with carbohydrate, protein and fat components of the HM. However, the ratio of energy from carbohydrate and fat of dietary intake of macronutrients, and breastfeeding assessment had significant effects of 9% as R^2^ = 0.09 on HM energy. That is, the higher the ratio of energy consumed from carbohydrates (β = 0.86, *p* < 0.01), fat (β = 0.77, *p* < 0.05) from dietary intake of macronutrients, and the higher the breastfeeding assessment score (β = 0.17, *p* < 0.05), the higher the HM energy (Table 4).

**Figure 1:**
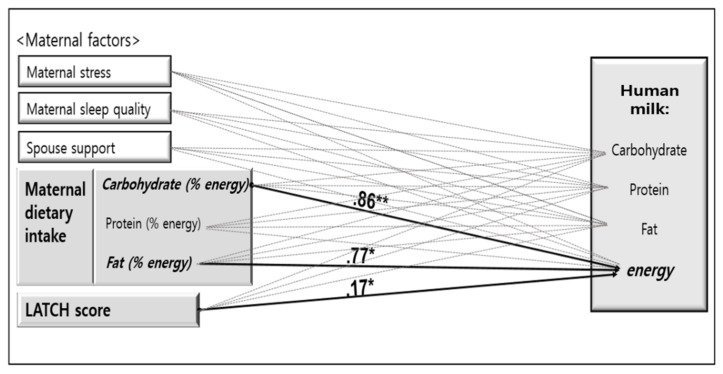
Graphical description of the human milk influencing factors. The dotted lines indicate a statistically insignificant association, whereas the solid lines indicate a statistically significant association. Values above the solid lines indicate the correlation coefficients drawn from the regression. * *p* < 0.05; ** *p* < 0.01, *p*: *p*-value.

The overall study results are summarized below. Solid lines indicate a significant association between the variables, and dotted lines indicate an insignificant association (Figure 1).

## 4. Discussion

The most important finding of this study was that psychosocial factors such as stress, sleep quality, and spousal support of postpartum breastfeeding mothers were not significantly related to HM macronutrients and energy, whereas the dietary intake of macronutrients and breastfeeding assessment factors were found to be significantly related.

First of all, the results demonstrated that postpartum stress, sleep quality, and spousal support were not related to HM macronutrients and energy. This was in line with the findings in Soffer et al. who, by repeatedly observing breastfeeding mothers’ anxiety and HM macronutrient content at weekly intervals, found that the psychological anxiety of mothers did not affect the HM macronutrient content [25]. Nevertheless, Soffer et al. identified only the anxiety factors of breastfeeding mothers in relation to the HM macronutrients of 21 participants. Along with previous studies that did not find significant relationships between psychological factors such as stress in breastfeeding mothers and HM immune components, such as Aparicio et al. [30], the results of this study add evidence to the possible existence of a buffering mechanism that maintains the homeostasis of HM macronutrients regardless of the body’s physiological adaptation process to internal and external stimuli, such as a mother’s stress, in milk production.

The results of this study can be used as evidence that the psychological aspects of breastfeeding mothers have a limited effect on HM macronutrients and energy, even if the mothers in the postpartum period are experiencing psychological difficulties. Lyons et al. reported that belief in the nutritional adequacy and sufficiency of HM promoted breastfeeding behavior [31]. Unlike food intake that one can control, the stress or sleep quality that breastfeeding mothers experience in the process of adapting to the maternal role cannot be arbitrarily controlled, which may potentially reduce the mother’s desire to breastfeed or interfere with the continuation of lactation. Therefore, the results support psychological interventions for breastfeeding promotion and continuance.

Second of all, the ratio of energy intake from carbohydrates and energy intake from fat among dietary intake of macronutrient subcategories showed significant quantitative relationships with HM energy. This is in line with the results in Bzikowska-Jura et al. that showed a positive correlation between the ratio of energy intake from protein and HM energy, as well as a positive correlation between the ratio of energy intake from carbohydrates and HM fat, in that dietary macronutrient intake showed a significant association with HM energy and macronutrients [32]. There was also a similarity with research reporting that the lipid content could change according to the dietary intake [4,33]. In terms of HM carbohydrates, the study result that it had no association with the factors of breastfeeding mothers, was similar to the result of a previous study in which carbohydrate content was largely maintained during the lactation period [34]. However, the results of this study differed from those reporting that the quantity or quality of HM was not affected by the nutritional status and dietary intake of breastfeeding mothers [10,12,35], or that there was little effect of the mother’s dietary intake on HM lipids [36].

Third of all, breastfeeding assessment represented as LATCH scores showed significant positive relationships with HM energy. Breastfeeding assessment was previously shown to be positively related with breastfeeding duration [21,37,38], breastfeeding volume [39], and infant weight gain [40], and studies such as Butte et al. show that infant weight is associated with higher fat and total energy intake [35]. This requires further investigation into the specific impact channels of breastfeeding assessment. In this sense, this study examined the direct effect of breastfeeding assessment on the HM composition itself. The study result that higher LATCH scores were associated with higher HM energy fills the gap in previous studies on the impact of breastfeeding assessment and provides evidence to the importance of breastfeeding assessment and the provision of nursing intervention for postpartum breastfeeding assessment. In addition to tackling the difficulties of breastfeeding, the value and function of breastfeeding assessment may be extended to affecting the HM energy, and therefore, the growth of babies. This association can be used as scientific foundations for developing nursing interventions to increase breastfeeding rates, providing further reassurance to breastfeeding mothers of the HM content and energy value.

In order to further verify the effects of dietary factors and breastfeeding assessment on HM macronutrients in postpartum breastfeeding mothers, additional research such as a longitudinal study with a period of at least 6 months after childbirth, the recommended period of exclusive breastfeeding by the World Health Organization, are needed.

There are several limitations to this study. First, it is a cross-sectional study that does little to verify the “causal” relationship between the variables examined. Second, the study conveniently sampled a limited number of breastfeeding mothers in postpartum care facilities from a limited regional scope. It may be difficult to generalize the results of this study on the relationship with macronutrients to all breastfeeding mothers outside of Korea. In particular, the postpartum care centers that are widely used by mothers in Korea from which the study participants were sampled provide them with the same daily diet at equal hours as well as with various types of assistance, from 24-h newborn care services to body massages for relaxation and recovery. Third, this study analyzed the components of ‘transitional human milk,’ which indicates HM formed during a specific period after childbirth. Therefore, since the results of this study confirm the influence of maternal factors on transitional milk macronutrient components, the implication of the results is limited for the relationship between maternal factors and HM components in colostrum and mature milk. This study examined human milk at the transitional stage in order to best control for external conditions such as the lactation stage and postpartum living environment. It was possible to gather over 150 study participants at exactly 1–2 weeks after childbirth thanks to the nationwide use of postpartum care facilities in Korea. However, in terms of colostrum or mature milk, there were serious practical difficulties in gathering the samples. In the case of colostrum milk, most breastfeeding mothers in this study experienced insufficient secretion of colostrum to provide their babies, making it difficult to obtain colostrum samples for study. In the case of mature milk, it was realistically challenging to gather a considerable number of breastfeeding mothers that were in similar stages of the lactation period, making it difficult to implement a study with a vast group of participants. Fourth, this study analyzed human milk components using a US FDA-certified Semisolid-state mid-infrared analyzer which could only measure the macronutrient (carbohydrate, fat, protein) quantity and energy. Under the circumstances, this study primarily found its value in verifying the relationship of the human milk with macronutrient intake through the breastfeeding mother’s diet. Hence, future research should further investigate the relationship between human milk components and a breastfeeding mother’s diet and various other nutrients that may affect the growth and development of the baby—arachidonic fatty acids, EPA and DHA, vitamins D, K, and C, etc.

The findings in this study are meaningful in that they identify some of the potential causes of the considerable variations found among the HM macronutrient content among breastfeeding mothers. There were not only large variations in the HM macronutrient content among the study participants, but also the average HM component of the study participants was lower in protein and higher in fat than in other previous studies [35,41]. If the HM content is excessive or insufficient, it may affect the health of the infant. Furthermore, this study was based on a large number of participants—159 breastfeeding mothers. A study of this volume is hard to come across in previous literature, not to mention that it is the first attempt made among Korean breastfeeding mothers. Lastly, the study considered multiple potential factors that may affect the HM macronutrients including dietary intake, breastfeeding assessment, psychosocial factors such as stress, sleep quality, and spousal support. This allows for a more comprehensive overview of the impact factors of human milk.

## 5. Conclusions

This study examined the effect of postpartum maternal factors on HM macronutrients and energy. Among the maternal factors, the dietary intake of macronutrient and breastfeeding assessment factors were significantly related to HM energy. The ratio of energy intake from carbohydrates and the ratio of energy intake from fat showed a significant quantitative relationship with HM energy. Breastfeeding assessment using the LATCH tool was also found to have an impact on the energy of HM, suggesting that the benefits of breastfeeding assessment are not limited to improving breastfeeding difficulties, but are extended to the HM quality itself, such as an increase in HM energy value.

On the other hand, the postpartum stress, sleep quality, and spousal support of breastfeeding mothers did not significantly affect HM macronutrients. This may be the basis for showing that the homeostasis of HM macronutrients is maintained against variations in psychosocial factors, or the body’s basic physiological action against stress.

Through these results, this study expands the knowledge of human milk components, providing necessity for psychological intervention as well as dietary education and breastfeeding assessment (LATCH) for the health of breastfeeding mothers and babies, and providing the basis for intervention development in consideration of the participants’ cultural characteristics.

## Figures and Tables

**Table 1:** General characteristics of participants (*n* = 159).

Variable	Categories		Frequency	Percent
Education	high school or lower		18	11.3
	college or higher		141	88.7
Job	not working		87	54.7
	working		72	45.3
Monthly income (1000 won)	less than 3000		66	41.5
	3000 and over		93	58.5
Maternal age (years)	Min = 21, Max = 45, Mean ± SD = 33.98 ± 4.35			
Parity (times)	1		106	66.7
	2		41	25.8
	3		12	7.5
Delivery type	normal		87	54.7
	C/S		72	45.3
Feeding type	exclusive breastfeeding		32	20.1
	mixed feeding		127	79.9
Breastfeeding education	experienced		115	72.3
	not experienced		44	27.7
Breastfeeding interval (hours)	sometimes		9	5.7
	1~2		19	11.9
	2~3		68	42.8
	3~4		50	31.4
	over 4		13	8.2
Number of breastfeeding per day (times)	1–4		44	27.7
	5–8		101	63.5
	9–12		14	8.8
The first reason for Breastfeeding choice	benefits of breastfeeding		88	55.3
	family’s recommendation		1	0.6
	own will		65	40.8
	expert’s advice		4	2.5
	others		1	0.6
The person who most influenced breastfeeding decision	own will		75	47.2
	husband		31	19.5
	mother		43	27.0
	doctors		1	0.6
	nurses		5	3.1
	others (parents in law, friends etc.)		4	2.5
	don’t know		19	11.9
One breastfeeding amount	lack		1	0.6
	appropriate		74	46.7
	excessive		55	34.3
	no response		11	6.5
BMI (kg/m^2^)	BMI-pre Min = 15.63, Max = 31.25 Mean ± SD = 21.66 ± 2.54			
	BMI-full	Min = 20.45, Max = 34.89, Mean ± SD = 26.82 ± 2.57		
	BMI-present	Min = 9.00 Max= 31.99, Mean ± SD = 24.16 ± 2.73		

BMI = Body mass index (kg/m^2^); BMI-pre = BMI before pregnancy; BMI full = BMI at full term; BMI-present = current BMI; C/S = Cesarean section.

**Table 2:** Descriptive statistics of the main variables (*n* = 159).

Variables		Min	Max	Mean ± SD	Skewness	Kurtosis
Stress		9.00	27.00	18.54 ± 4.21	0.01	−0.49
Sleep quality		13.00	97.00	55.81 ± 18.37	−0.10	−0.74
Spouse support		25.00	125.00	105.56 ± 15.97	−1.30	3.31
Dietary intake of macronutrients (%E)	carbohydrate	35.00	79.00	56.90 ± 0.10	−0.13	−0.08
	protein	9.00	28.00	15.90 ± 0.04	0.99	1.24
	fat	10.00	42.00	26.30 ± 0.07	−0.12	−0.10
Breastfeeding assessment (LATCH)	latch	0.00	2.00	1.30 ± 0.62	−0.30	−0.63
	audible swallowing	0.00	2.00	1.47 ± 0.59	−0.63	−0.55
	nipple type	0.00	2.00	1.62 ± 0.60	−1.36	0.81
	level of comfort	0.00	2.00	1.63 ± 0.53	−1.04	0.12
	help	0.00	2.00	1.62 ± 0.54	−0.97	−0.13
Macronutrients of HM	carbohydrate (g/dL)	5.70	7.40	6.64 ± 0.27	−0.42	1.46
	protein (g/dL)	0.50	2.40	1.32 ± 0.25	−0.01	3.17
	fat (g/dL)	0.80	7.90	3.45 ± 1.28	0.74	0.55
	energy (kcal/dL)	33.00	102.00	63.18 ± 11.22	0.49	0.49

%E = %energy; Min = minimum; Max = maximum; SD = standard deviation; LATCH is a breastfeeding charting system that provides a systematic method for gathering information about individual breastfeeding sessions. LATCH = ‘L’ latch, ‘A’ Audible swallowing, ‘T’ nipple type, ‘C’ level of comfort, ‘H’ help (Jensen et al., 1994). The percentage of energy obtained from each macronutrient is calculated by allocating carbohydrates, protein, and fat 4 kcal/g, 4 kcal/g, 9 kcal/g, respectively. The specific formulas used are as follows: (i) Carbohydrate %E = (carbohydrate × 4)/(total energy intake per day) × 100, (ii) Protein %E = (protein × 4)/(total energy intake per day) × 100, (ⅲ) Fat %E = (fat × 9)/(total energy intake per day) × 100.

**Table 3:** Pearson’s correlation analysis of postpartum stress, sleep quality, spousal support, dietary intake of macronutrients, breastfeeding assessment, and HM macronutrients and energy (*n* = 159).

Variables		Stress	Sleep Quality	Spousal Support	Dietary Intake of Macronutrients(%E)			LATCH	HM Macronutrients			
					Car.	Fat	Pro.		Fat (g/dL)	Pro. (g/dL)	Car. (g/dL)	Energy (kcal/dL)
Stress		1										
Sleep quality		0.34 **	1									
Spousal support		−0.35 **	−0.16 *	1								
Dietary intake of macronutrients (%E)	Car.	0.00	−0.04	0.13	1							
	fat	0.03	0.07	−0.11	−0.96 **	1						
	Pro.	−0.01	−0.04	−0.16 *	−0.86 **	0.73 **	1					
LATCH		−0.21 **	−0.08	0.26 **	−0.01	−0.01	−0.04	1				
Macronutrients of HM	fat (g/dL)	−0.05	0.08	0.02	0.11	−0.06	−0.17 *	0.14	1			
	Pro. (g/dL)	−0.09	−0.09	0.00	0.02	−0.04	0.06	−0.09	−0.10	1		
	Car. (g/dL)	0.02	0.02	−0.05	−0.08	0.09	0.05	−0.09	−0.27 *	0.21 *	1	
	energy (kcal/dL)	−0.03	0.06	0.05	0.14	−0.08	−0.22 *	0.16 *	0.95 *	0.02	−0.14	1

* *p* < 0.05; ** *p* < 0.01; Car. = carbohydrate: Pro. = protein: Car. (%E) = Energy % from carbohydrate intake: Pro. (%E) = Energy % from protein intake: fat (%E) = Energy % from fat intake: LATCH = Breastfeeding assessment tool. LATCH is a breastfeeding charting system that provides a systematic method for gathering information about individual breastfeeding sessions.

**Table 4:** Effects of independent variables on HM energy (*n* = 159).

Independent	Variable		B	SE	β	t	*p*
(constant)			−37.21	33.04		−1.13	0.262
Dietary intake of macronutrients		carbohydrate (%E)	104.31	35.14	0.86	2.97	0.004 **
		fat (%E)	127.31	48.45	0.77	2.63	0.010 *
Breastfeeding assessment			1.04	0.50	0.17	2.11	0.037 *

* *p* < 0.05; ** *p* < 0.01; Carbohydrate (%E) = Energy % from carbohydrate intake; fat (%E) = Energy % from fat intake. B: estimates, SE: standard error, β: standardized estimates, t: B/SE, *p*: *p*-value.
